# Functional Connectivity and Networks Underlying Complex Tool-Use Movement in Assembly Workers: An fMRI Study

**DOI:** 10.3389/fnhum.2021.707502

**Published:** 2021-10-28

**Authors:** Seira Taniguchi, Yuichiro Higashi, Hirotaka Kataoka, Hiroshi Nakajima, Tetsuya Shimokawa

**Affiliations:** ^1^Center for Information and Neural Networks, Advanced ICT Research Institute, National Institute of Information and Communications Technology, Suita, Japan; ^2^Omron Corporation, Kizugawa, Japan

**Keywords:** complex tool-use, assembly work, fMRI, functional connectivity, network, rehabilitation

## Abstract

The aim of this study was to identify the functional connectivity and networks utilized during tool-use in real assembly workers. These brain networks have not been elucidated because the use of tools in real-life settings is more complex than that in experimental environments. We evaluated task-related functional magnetic resonance imaging in 13 assembly workers (trained workers, TW) and 27 age-matched volunteers (untrained workers, UTW) during a tool-use pantomiming task, and resting-state functional connectivity was also analyzed. Two-way repeated-measures analysis of covariance was conducted with the group as a between-subject factor (TW > UTW) and condition (task > resting) as a repeated measure, controlling for assembly time and accuracy as covariates. We identified two patterns of functional connectivity in the whole brain within three networks that distinguished TW from UTW. TW had higher connectivity than UTW between the left middle temporal gyrus and right cerebellum Crus II (false discovery rate corrected *p*-value, *p*-FDR = 0.002) as well as between the left supplementary motor area and the pars triangularis of the right inferior frontal gyrus (*p*-FDR = 0.010). These network integrities may allow for TW to perform rapid tool-use. In contrast, UTW showed a stronger integrity compared to TW between the left paracentral lobule and right angular gyrus (*p*-FDR = 0.004), which may reflect a greater reliance on sensorimotor input to acquire complex tool-use ability than that of TW. Additionally, the fronto-parietal network was identified as a common network between groups. These findings support our hypothesis that assembly workers have stronger connectivity in tool-specific motor regions and the cerebellum, whereas UTW have greater involvement of sensorimotor networks during a tool-use task.

## Introduction

The unique ability to handle tools with high speed and accuracy for performing daily tasks and work can distinguish our species from non-human primates. Greater skills with manipulating tools and conceptual knowledge facilitate tool-use performance in humans (Liepmann, 2001; Koski et al., 2002). For instance, humans have more pronounced skills than chimpanzees in these cognitive domains of tool-use: fine motor control in hand–eye coordination, inferential causal reasoning, function representation, inhibition and foresight in executive control, teaching, time estimation in contingent reciprocity, and language (Vaesen, 2012).

Neuropsychological studies have revealed tool-specific regions in the brain, including the superior parietal lobule (SPL), ventral premotor cortex (PMv), and middle temporal gyrus (MTG), especially in the left hemisphere. Indeed, left hemisphere lesions involving the inferior parietal lobule cause “apraxia,” which is a clinical syndrome underscored by difficulties in performing learned and skilled gestures (Gross and Grossman, 2008). Additionally, “ideational apraxia” is a subtype of apraxia that refers to the inability to grasp and manipulate tools accurately on the basis of their perceptual properties (Buxbaum et al., 2003). The cerebellum is important for motor learning and control. Imamizu et al. (2000) identified that cerebellar activation occurs not only in the early phase of tool-use learning but also after the learning is complete. Yoo et al. (2013) have proposed that the fronto-parietal network is a core network underpinning skilled tool-use during the resting state. They reported that sensorimotor networks exhibited decreased connectivity after handling chopsticks (Yoo et al., 2013). However, they only investigated functional connectivity and networks in the resting state and did not assess task-related states during tool-use. Although brain activation studies have provided insight into the neural bases of human tool-use, the neural networks involved in the execution of professional tool-use in assembly workers have not been elucidated.

Moreover, neuroimaging studies have indicated an overlap in regions underscoring actual tool-use and pantomiming (Choi et al., 2001; Fridman et al., 2006; Buxbaum et al., 2014; Hoeren et al., 2014; Lausberg et al., 2015), mental imagery (Grèzes et al., 2003; Creem-Regehr and Lee, 2005), and action observation (Beauchamp et al., 2002; Johnson-Frey et al., 2003; Macdonald and Culham, 2015). Many neuroimaging paradigms have employed tasks involving pantomiming tool-use because it is thought to approximate the process of planning to use actual tools (Lewis, 2006). A meta-analysis revealed that the SPL, dorsolateral lobule, PMv, inferior parietal lobule, and MTG are selectively activated when pantomiming tool-use (Lewis, 2006). Nevertheless, most neuroimaging studies involving pantomiming employ relatively simple tasks, such as tool-use demonstrations with either the right or left hand (Choi et al., 2001; Fridman et al., 2006; Buxbaum et al., 2014; Hoeren et al., 2014; Lausberg et al., 2015). In contrast, the use of tools in real-life settings is more complex than that in experimental environments. For example, using a hammer and nails, scissors and papers, or screwdrivers and screws requires the use of both hands. Furthermore, performance may vary depending on the physical environment (Jovanovic et al., 2007), including the height of the platform and chair and the size or weight of tools, which may impact tool-use efficiency. Nevertheless, the brain networks underscoring complex tool-use performance in real-life environments have not been elucidated. The aim of the current study was therefore to investigate the characteristics of real assembly workers in a tool-use pantomiming task. We examined the functional connectivity and neural networks underlying tool-use movement in professional assembly workers, who perform complex tool-use in their daily work. We hypothesized that real assembly workers have stronger connectivity in the tool-specific regions and cerebellum than untrained workers (UTW) and that UTW have greater involvement of sensorimotor networks than trained workers (TW).

## Materials and Methods

### Ethical Approval

This study was approved by the Ethics Committees of National Institute of Information and Communications Technology (No. 1809070050) and was conducted in conformance with the Declaration of Helsinki. We obtained written informed consent from each participant after explaining the purpose, expected benefits, and potential harm of this research.

### Participants

We recruited 13 workers (mean age, 35.8 ± 10.5 years; range, 21–52 years old; 11 women and 2 men) from an assembly plant for factory automation parts owned by Omron Co. (henceforth referred to as TW), and 27 age-matched volunteers who had no experience with assembly tasks (mean age, 37.9 ± 10.0 years; range, 21–54 years old; 13 women and 14 men) *via* advertisements and consultation referrals (henceforth referred to as UTW).

The inclusion criteria were: (1) being able to precisely follow the process of the actual assembly task, including sequence errors and (2) having a normal or corrected-to-normal vision, and (3) having no contraindications for MRI.

### Behavioral Assessment

**FIGURE 1:**
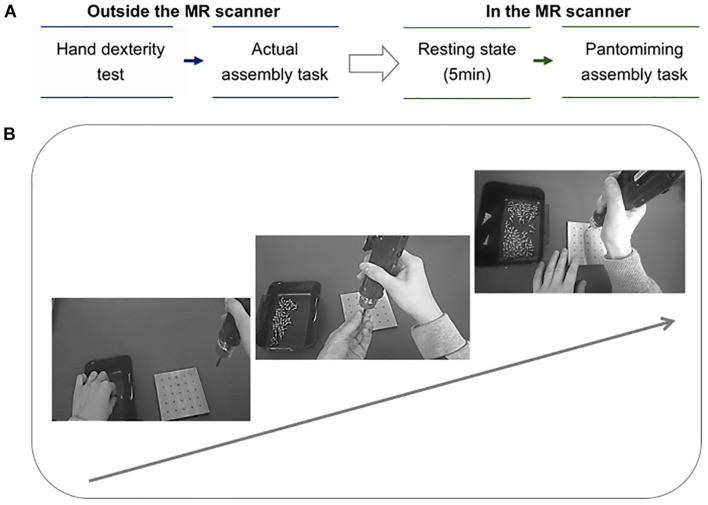
**(A)** Protocol summary. The arrow indicates the order of the tasks, from outside the MR scanner to inside the MR scanner. There are two phases in the protocol: (1) two tests were conducted outside the MR scanner. First, the participants performed the manual hand dexterity test using the Purdue Pegboard Test, and then they underwent the actual assembly task. (2) Imaging using the MR scanner. Second, the participants underwent resting-state fMRI for 5 min. Thereafter, an fMRI scan was executed as the participants were instructed to pantomime the assembly task while viewing their own performance. **(B)** Assembly task process. The arrow indicates the workflow of the assembly task. The participants were instructed to pick up a screw from the basket on the left with their left hand and attach it to the tip of the device, then insert the point of the screw into the hole of the square base. Finally, the thumb of the right hand was used to push the button on the handle of the device for tightening the screw. This process was repeated from the top hole of the base to the sixth hole.

The protocol summary and the assembly task process are presented in Figures 1A,B, respectively. Prior to fMRI scanning, participants underwent two behavioral sessions consisting of: (1) actual assembly task, and (2) manual hand dexterity test.

#### Actual Assembly Task

Participants were required to complete a screwing task as precisely as possible. For the display of the pantomiming task in the MR (magnetic resonance) scanner, the performance of each participant’s assembly task was recorded using a wearable camera (Tobii Technology, Danderyd, Sweden, Tobii Pro Glasses 2) with a video resolution of 1920 × 1080 at 25 fps. Performance time was measured with a stopwatch. Each participant practiced tightening screws in order to confirm each step of the task prior to scanning.

The index of “*accuracy*” of the assembly performance was defined by the number of mistakes while tightening screws, ranging from 0 to 6 errors. Zero errors were indicative of good accuracy, while higher errors were indicative of poor accuracy. The two investigators (ST and YH) independently viewed videos of participants’ performances to check whether each screw was fully seated in the base. If the shank remained above the surface of the base, the investigator counted this as a mistake. If there was a disagreement in numbers of missed tightening between the investigators, the final decision was made after a discussion.

#### Manual Hand Dexterity Test

This test involved the Purdue Pegboard Test (SAKAI Medical Co., Ltd., Tokyo, Japan, A929-1), which reflects quantitative hand dexterity function and speed ability, and finger–eye coordination (Backman et al., 1992). The test was originally designed to assess the manual dexterity of assembly line employees (Radomski and Trombly, 2008).

### MRI Scanning

Participants viewed a display while lying supine in the MRI bore. During scanning, participants were instructed to pantomime the assembly task (a simple block design) as precisely as possible without physically interacting with tools. The participants were asked to continuously view the video sequences showing their own performance on the screen at a distance of around 130 mm, using a mirror attached to a head coil. Resting-state fMRI runs of 5-min were performed immediately prior to fMRI runs of tasks, during which all participants were instructed to avoid repetitive thoughts. Thin foam pillows were positioned around each participant’s head to minimize head motions.

### MRI Acquisition and Pre-processing

MRI scans were collected using a 64-channel head coil and MAGNETOM Vida 3T scanner (Siemens, Erlangen, Germany) at the Center for Information and Neural Networks (Osaka, Japan). Functional data were obtained using a T2^∗^-weighted echo-planar imaging (EPI) pulse sequence with the following parameters: repetition time (TR)/echo time (TE) = 1000/30 ms, flip angle = 60°, 39 slices, matrix size = 3.0 mm × 3.0 mm × 4.0 mm, field-of-view (FoV) = 192 mm, slice thickness = 4.0 mm, and an interleaved mode of slice acquisition. For the T1-weighted data, EPI employed the following parameters: TR/TE = 3.15/1.37 ms, 300 volumes, 39 slices, flip angle = 8°, voxel size = 1.6 mm × 1.6 mm × 1.6 mm, FoV = 260 mm, slice thickness = 1.6 mm.

Functional and structural MRI data were pre-processed using CONN toolbox version 17.f^1^ implemented in MATLAB (MathWorks Inc., Natick, MA, United States). On pre-processing, the data were realigned, unwarped, slice-timing corrected, co-registered to each individual T1 anatomical image, spatially normalized to Montreal Neurological Institute space, outlier detected (ART-based scrubbing), and smoothed with 8-mm full-width-at-half maximum Gaussian kernel. Subsequently, a denoising pipeline was performed by implementing the aCompCor strategy (Behzadi et al., 2007), including linear regression (regression of noise variables derived from motion, cerebrospinal fluid, and white matter) and band-pass filtering (0.008–0.09 Hz) (Nieto-Castanon, 2020).

Participants were excluded if excessive head movement determined by the mean framewise displacement (FD) was more than 0.5 mm (Power et al., 2014), which revealed no between-group difference in mean FD between TW and UTW in the pantomiming task (TW = 0.170 ± 0.038 vs. UTW = 0.192 ± 0.055, *p* = 0.31) and resting-state (TW = 0.138 ± 0.042 vs. UTW = 0.158 ± 0.048, *p* = 0.28).

### ROI-to-ROI Analysis

The commonly used approach to analyze task-related fMRI, such as psychophysiological interaction (PPI) and dynamic causal modeling (DCM); however, they usually implicitly assume that specific functional connectivity linked to a task exists. To the best of our knowledge, no report regarding important functional connectivity or regions or networks linked to complex assembly tool-use exists. Therefore, we applied region-of-interest (ROI)-to-ROI analysis to identify functional connectivity in TW, as a precursor to PPI or DCM analysis.

Recent studies used ROI-to-ROI analysis to identify specific functional connectivity in martial arts performers (Fujiwara et al., 2019) and in people with visual snow (Aldusary et al., 2020).

Region-of-interest-to-ROI connectivity matrices were computed for each subject and for each source ROI in the first-level analysis. A 264 × 264 matrix for each participant was defined as the Fisher transformed bivariate correlation coefficients for each pair of the 264 regions. ROIs were labeled based on the Power et al. (2011) atlas (Power et al., 2011) and were divided into eight networks: somatomotor hand network (SMN) with 30 ROIs, visual network with 31 ROIs, auditory network with 13 ROIs, default mode network (DMN) with 58 ROIs, fronto-parietal network (FPN) with 25 ROIs, cingulo-opercular network with 14 ROIs, dorsal attention network with 11 ROIs, and ventral attention network (VAN) with 9 ROIs.

The weighted general linear model used for the second-level analysis. Two-way repeated-measures analysis of covariance (ANCOVA) was conducted with the group as a between-subject factor (TW > UTW) and condition (task > resting) as a repeated measure, controlling for assembly time and accuracy as covariates. *Post hoc* two-sample *t*-tests were performed for ROIs with significant group × condition interactions. Significant connections were identified by calculating the false discovery rate (FDR) corrected with two-sided *p*-values < 0.01.

### Statistical Analysis

Between-group differences in hand dexterity test scores and time of actual assembly task were assessed using the Mann–Whitney *U* test. The scores of accuracy in the assembly task and gender differences between groups were assessed using the Chi-squared test. The correlation between identified functional connectivity and other variables of assembly task performance was assessed using Spearman’s rank correlation coefficient (Rs).

## Results

### Participant Characteristics and Behavioral Results

Participant characteristics are presented in Table 1. TW had a larger proportion of females than that of UTW (*p* < 0.05) and exhibited significantly higher speed in the assembly task (*p* < 0.001). Similar group differences were observed in assembly task accuracy (*p* = 0.146) and manual hand dexterity test scores (*p* = 0.568). No participant exceeded the mean FD value of 0.5 mm.

**TABLE 1:** Participants’ characteristics.

	Trained workers (*n* = 13)	Untrained workers (*n* = 27)	*T* statistics and df	*p*-values
Age (years)	35.8 ± 10.5	37.9 ± 10.0	*F*(1,38) = 0.36	*p* = 0.648
Gender (female/male)	11/2	13/14	χ^2^ = 0.03 (φ = −0.35)	*p* < 0.05
Assembly time (s)	23.7 ± 3.4	44.3 ± 10.8	*F*(1,38) = 44.26	*p* < 0.001
Accuracy (%)*	3 errors = 0%	3 errors = 3.7%	χ^2^ = 5.38 (φ = 0.38)	*p* = 0.146
	2 errors = 0%	2 errors = 14.8%		
	1 error = 16.7%	1 error = 33.3%		
	0 error = 83.3%	0 error = 48.1%		
Manual hand dexterity: pegboard test (s)	38.5 ± 6.7	37.4 ± 5.1	*F*(1,38) = 0.33	*p* = 0.568

*Number of mistakes while tightening screws (range 0–6 screws = % of subjects within the group).

### Specific Functional Connectivity for Assembly Tool-Use in Trained Workers and Untrained Workers

**FIGURE 2:**
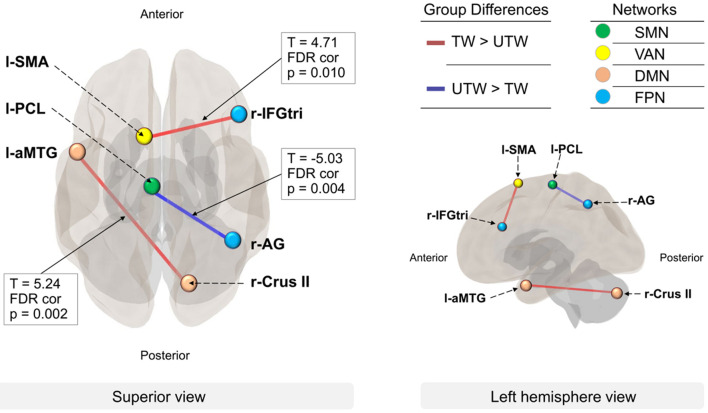
Between-network connectivity in TW and UTW. The lines represent the functional connectivity with other ROIs (FDR-corrected, *p* < 0.01). Group differences: red lines represent higher connectivity in TW than in UTW (TW > UTW); blue lines represent higher connectivity in UTW than in TW (UTW > TW). Networks: colored dots indicate the four networks with significance at *p* < 0.01. Green, somatomotor hand network (SMN); yellow, ventral attention network (VAN); pink, default mode network (DMN); light blue, fronto-parietal network (FPN). ROIs: l-aMTG, left anterior middle temporal gyrus; r-Crus II, right cerebellum crus II; l-SMA, left supplementary motor area; r-IFGtri, pars triangularis of the right inferior frontal gyrus; l-PCL, left paracentral lobule; r-AG, right angular gyrus.

Results of the whole-brain analysis are presented in Figure 2.

For better identification of connectivity during the assembly task, the thresholds for ROI-to-ROI connectivity in the whole brain analysis were increased to *p*-FDR < 0.01.

At the ROI level for group comparisons of functional connectivity, as compared with UTW, TW had increased connectivity between the left MTG and right cerebellum Crus II [*T*(36) = 5.24, *p*-FDR = 0.002], which were components of the DMN, as well as between the left supplementary motor area (SMA) and the pars triangularis of the right inferior frontal gyrus (IFGtri) of Broca’s area [*T*(36) = 4.71, *p*-FDR = 0.010], which are components of the VAN and FPN, respectively. In contrast, UTW showed a stronger integrity compared to TW between the left paracentral lobule (PCL) and right angular gyrus (AG) [*T*(36) = −5.03, *p*-FDR = 0.004], which are components of the SMN and FPN, respectively.

### Relationship Between Identified Functional Connectivity and Assembly Task Performance

The relationship between identified functional connectivity and the scores of assembly task performance, and the relationship between assembly time and identified functional connectivity is illustrated in a scatterplot.

Assembly time was inversely correlated with functional connectivity between the MTG and Crus II in all the participants, indicating a higher speed in the assembly task related to stronger connectivity between the MTG and Crus II. The UTW’ slower assembly time correlated with a stronger TW-specific connectivity, while a faster assembly time was only related to a stronger UTW-specific connectivity. Further, there was no significant relationship between the scores of accuracy and functional connectivity.

## Discussion

### Behavioral Results

Our study is the first to demonstrate the functional connectivity and associated networks among 264 nodes underscoring skillful tool-use performance in assembly workers in a real tool-use environment by investigating the differences between TW and UTW. TW exhibited greater speed in the assembly task, although no group differences were observed in assembly task accuracy or manual hand dexterity. These findings suggest that complex tool-use ability and hand dexterity are distinct in relation to their neural substrates.

As for the gender differences in this study, a recent systematic review by Hirsch et al. (2019) summarized that there were no gender differences in both sequential and concurrent multitasking conditions. This may support the gender imbalance of this study group that might not have greatly influenced our results in the assembly task.

### Functional Connectivity and Networks

Two patterns of functional connectivity between anterior MTG and Crus II, and between SMA and IFGtri were TW-specific, while that between PCL and AG was UTW-specific. Moreover, our results from the correlation analysis would further support these characteristic findings.

At the network level, the DMN and the VAN were TW-specific, while the SMN was UTW-specific. Additionally, the FPN was identified as a common network between groups.

#### Trained Workers Specific Functional Connectivity Between Anterior Middle Temporal Gyrus and Crus II

The posterior MTG has consistently been reported to be involved in tool-use (Lewis, 2006), whereas, in this study, the MTG was located anteriorly (*x*: −53, *y*: 3, *z*: −27), which indicated that the anterior activity was more strongly associated with frequently used tools than with rarely used tools (Vingerhoets, 2008) and was a key region for the retrieval of stored semantic information (Xu et al., 2015). Goodale et al. (1994) suggested the pantomimed task was elicited by stored perceptual information even if the object was visually present, while actual grasping relies on the visuo-motor online control system. Collectively, these findings indicate that the anterior MTG is involved in retrieving conceptual knowledge about familiar tools in TW.

Interestingly, the cerebellar Crus II was identified as a previously unreported region associated with tool-use. Crus II has been often referred to as the “cognitive cerebellum” (Buckner et al., 2011). For example, retrieval of episodic memories (Huo et al., 2018) and verbal working memory (Diedrichsen and Zotow, 2015). Similarly, the DMN is involved in working memory (Piccoli et al., 2015; Sormaz et al., 2018). However, retroviral tracing studies have revealed that the Crus II is also related to motor functions (Kelly and Strick, 2003; Greger et al., 2004). Another study showed that the Crus II contributes to the accurate temporal prediction of absolute timing in voluntary movement tasks (Yamaguchi and Sakurai, 2016) and short-term prediction through internal modeling (Lesage et al., 2017). Based on the cerebellar internal model theories, a feedforward control in the motor control system acts for relatively fast movements, while a feedback control is crucial for unskilled movement (Ghez et al., 1990). Thus, the rapid performance of TW during the pantomiming task may reflect a feedforward motor control by the cerebellum internal model.

Additionally, the faster assembly time was related to stronger connectivity between the anterior MTG and Crus II during the pantomiming task. This result suggests that rapid performance indicates a stronger neural network integrity between the anterior MTG and Crus II.

In summary, these results would suggest that familiarization of the tool-use related performance and a feedforward motor control are the key components for rapid tool-use in TW, underscored by the stronger network integrity between anterior MTG and Crus II.

#### Trained Workers Specific Functional Connectivity Between Supplementary Motor Area and Pars Triangularis of Inferior Frontal Gyrus

Although most SMA neuroimaging studies focus on motor planning and execution and IFGtri (Broca’s area) focus on linguistic function, the recruitment of SMA (Choi et al., 2001; Vingerhoets et al., 2011) and IFGtri (Higuchi et al., 2009) during tool-use related performance has been reported.

Importantly, both SMA and IFG are parts of mirror neuron system and such mirror neuron system has been demonstrated to be related to motor imagery, action recognition, and on-line imitation (Buccino and Riggio, 2006). This is widely known as functionally equivalent hypothesis (Jeannerod, 1994, 2001) since they have many similarities at a neural basis. In addition, considering the nature of the pantomiming task of this study that is accompanied by actual movements, recently proposed “dynamic motor imagery” (Schuster et al., 2011; Fusco et al., 2016, 2019) seems to be more related to the pantomiming task than conventional static motor imagery.

Furthermore, an fMRI study showed that IFG was activated in only the condition of finger motion imitation while that of hand motion did not, because there is a greater reliance on visual rather than sensory feedback in a finger imitation (Tanaka and Inui, 2002).

Moreover, at the network level, a study showed that VAN is involved in reorientation of attention as well as top-down and attentional capture processes (Solis-Vivanco et al., 2021). Thus, the recruitment of VAN may reflect cognitive loads in attention processing during the pantomiming task that involved sequential and repetitive tool-use movement.

Collectively, these findings suggested the superior ability of mental rehearsing by dynamic motor imagery is related to executing motor programs of the tool-use performance in a top-down manner, therefore, this connectivity was the TW’ specific during the assembly task. As for vocational rehabilitation, dynamic motor imagery training especially for fingers may be effective in the later stages of motor learning.

#### Untrained Workers Specific Functional Connectivity Between Paracentral Lobule and Angular Gyrus

The PCL is considered to be a combined motor and sensory region, primarily associated with the leg motor region (Baker et al., 2018). However, it remains unclear how the PCL is involved in manual movements in tool-use since the PCL has not been studied extensively in the context of tool-use compared to other regions. Further research is warranted to elucidate how this region is involved in assembly work. In addition, the AG is vital for perceptual sequence learning (Rosenthal et al., 2009) and finger recognition (Perry, 2014). Further, the AG is considered a cross-modal hub that merges multisensory information (Seghier, 2013). Indeed, Grèzes and Decety (2002) proposed that sensorimotor experience may be important for processing information to acquire access to semantic knowledge for certain classes of objects.

At the network level, the SMN was identified as being UTW-specific. Unsurprisingly, the somatosensory system contributes more strongly compared to the motor system in the early stages of motor learning (Bernardi et al., 2015).

Supporting these findings, the observed connectivity and network suggest that UTW may rely more heavily on sensory information to understand tool functions in order to acquire complex tool-use ability, similar to the development of tool-use skills in young children. As such, sensory discrimination training for hands may be effective for vocational rehabilitation in the early stage of learning.

#### Commonality Between Trained Workers and Untrained Workers: Fronto-Parietal Network

The FPN was a significant network for both TW and UTW groups. Neuroimaging studies have indicated that the FPN is involved in the processing of tools. FPN activation has been reported for both prospective and retrospective cues (Wallis et al., 2015). A positron emission tomography study revealed that the IFGtri and inferior parietal lobule were activated during the perception of tools, regardless of the tasks (Grèzes and Decety, 2002). This seems to contradict a specific role for these regions in tool-use; however, a recent review by Marek and Dosenbach (2018) suggested that “the frontoparietal network is a functional hub for influencing brain-wide communication to meet task demands.” This may explain involvement of the FPN during the assembly work in both TW and UTW.

### Limitations and Future Research

Several limitations of the current study should be acknowledged. First, although the sample size of the present study involving healthy subjects was greater than that of previous studies on tool-use (Choi et al., 2001; Beauchamp et al., 2002; Grèzes et al., 2003; Johnson-Frey et al., 2003; Creem-Regehr and Lee, 2005; Fridman et al., 2006; Vingerhoets et al., 2011; Yoo et al., 2013; Lausberg et al., 2015; Macdonald and Culham, 2015), more participants are necessary to draw more definitive conclusions. Second, data on factors influencing tool-use performance in participants, such as years of experience in assembly work, education, hobbies, and lifestyle, were not collected due to the company’s privacy policy. Third, our study adopted a cross-sectional design of two groups. A longitudinal study design may be more appropriate to investigate changes in tool-use learning over time. Furthermore, the supine position might have indeed influenced their pantomiming performance as they usually perform task in upright or sitting positions in real environments. However, we primarily focused on group differences between TW and UTW, having normalized by the same experimental setting. Moreover, although we focused on identifying the functional connectivity in TW by using ROI-to-ROI analysis, as a precursor to PPI or DCM analysis, further analysis should be performed to investigate the task-specific effect on the identified functional connectivity. Finally, from the scatterplot it appears that the TW had a relatively narrow range of distribution in assembly time compared to the UTW; therefore, it could not provide sufficient power for confirming the relationship with identified functional connectivity in TW. Larger samples within the TW group will enable further in-depth assessment of the relationship of functional connectivity with assembly time. Future studies should aim to address these limitations.

## Conclusion

We investigated the characteristics of real assembly workers in a tool-use pantomiming task to identify the functional connectivity and networks underlying tool-use movement. We identified two patterns of functional connectivity in the whole brain that distinguished TW from UTW, and a common network between the groups. TW had two stronger functional connectivity, between MTG and Crus II and between SMA and IFGtri. These network integrities may allow for TW to perform rapid tool-use. Notably, the Crus II was identified as a previously unreported region associated with tool-use. In contrast, UTW exhibited a stronger integrity between PCL and AG, which may reflect a greater reliance on sensorimotor input to acquire complex tool-use ability than that of TW. At the network level, the fronto-parietal network was was identified as a common network between groups. Further longitudinal studies with larger sample sizes as well as investigation of task-specific effects on the identified functional connectivity are required to confirm our findings.

## Data Availability Statement

The datasets presented in this article are not readily available because of ethical restrictions. Requests to access the datasets should be directed to ST, seira.taniguchi.pt@gmail.com.

## Ethics Statement

The studies involving human participants were reviewed and approved by the Safety Committee of National Institute of Information and Communications Technology (NICT). The participants provided their written informed consent to participate in this study.

## Author Contributions

HK, YH, and HN contributed to conception of the study. TS contributed design of the study. TS and ST organized the database. ST performed the statistical analysis and wrote the manuscript. All authors contributed to manuscript revision, read, and approved the submitted version.

## Conflict of Interest

YH, HK, and HN were employed by the company Omron Corporation. The authors declare that this study received funding from Omron Corporation (Kyoto, Japan). The funder was not involved in the study design, collection, analysis, interpretation of data, the writing of this article or the decision to submit it for publication.

## Publisher’s Note

All claims expressed in this article are solely those of the authors and do not necessarily represent those of their affiliated organizations, or those of the publisher, the editors and the reviewers. Any product that may be evaluated in this article, or claim that may be made by its manufacturer, is not guaranteed or endorsed by the publisher.

## Footnotes

1
www.nitrc.org/projects/conn

## Supplementary Material

Supplementary Figure 1 Correlation between the assembly time and identified functional connectivity. Scatterplots show the relationship between assembly time (*y*-axes) and identified functional connectivity (*x*-axes) and indicate Fisher’s *z*-values of functional connectivity between MTG and Crus II (top figure), SMA and IFGtri (middle figure), and PCL and AG (bottom figure). The box in black line encloses TW-specific connectivity, while the box in gray line encloses UTW-specific connectivity. The blue dots indicate the values in UTW, whereas the orange squares represent the values in TW. Significant results are shown in bold font. ^∗^*p* < 0.05, ^∗∗^*p* < 0.01. Rs, Spearman’s rank correlation coefficient; Total, all participants; TW, trained workers; UTW, untrained workers; MTG, left middle temporal gyrus; Crus II, right cerebellum Crus II; SMA, left supplementary motor area; IFGtri, pars triangularis of the right inferior frontal gyrus; PCL, left paracentral lobule; AG, right angular gyrus.

Supplementary Table 1 Spearman’s correlation coefficient between identified functional connectivity and the scores of assembly task performance.
