# Supplementary figures and images for: Functional Connectivity and Networks Underlying Complex Tool-Use Movement in Assembly Workers: An fMRI Study
Source: Front Hum Neurosci. 2021 Oct 28;15:707502. doi: 10.3389/fnhum.2021.707502 (PMC8581229; doi:10.3389/fnhum.2021.707502)

### TW-Specific connectivity

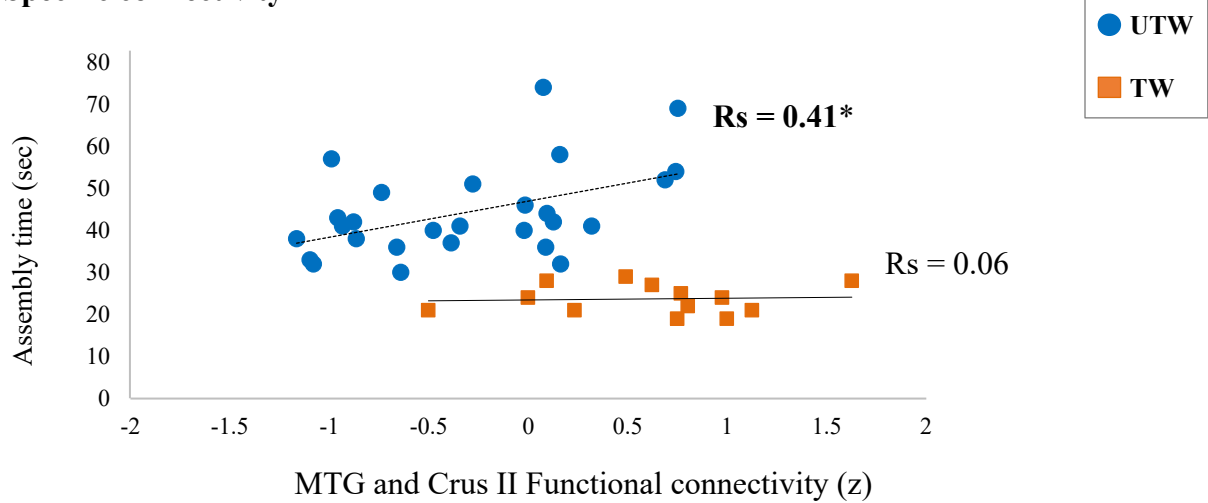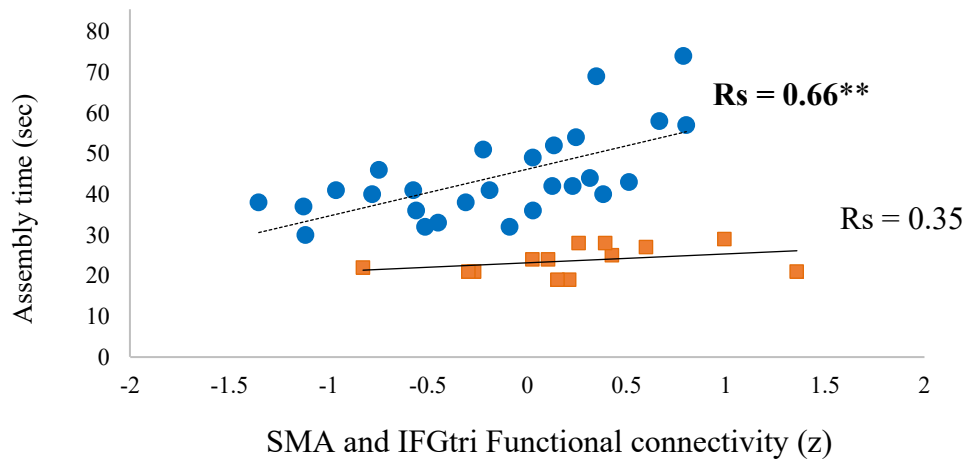

### UTW-Specific connectivity

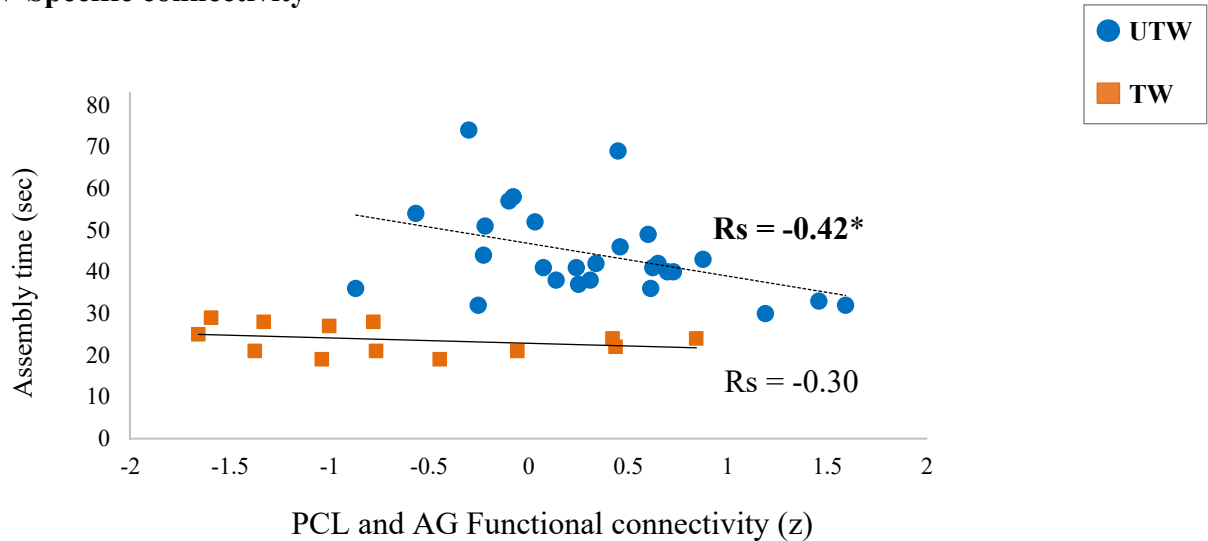

Supplement: Supplementary Figure 1 — Correlation between the assembly time and identified functional connectivity. Scatterplots show the relationship between assembly time (y-axes) and identified functional connectivity (x-axes) and indicate Fisher’s z-values of functional connectivity between MTG and Crus II (top figure), SMA and IFGtri (middle figure), and PCL and AG (bottom figure). The box in black line encloses TW-specific connectivity, while the box in gray line encloses UTW-specific connectivity. The blue dots indicate the values in UTW, whereas the orange squares represent the values in TW. Significant results are shown in bold font. ∗p < 0.05, ∗∗p < 0.01. Rs, Spearman’s rank correlation coefficient; Total, all participants; TW, trained workers; UTW, untrained workers; MTG, left middle temporal gyrus; Crus II, right cerebellum Crus II; SMA, left supplementary motor area; IFGtri, pars triangularis of the right inferior frontal gyrus; PCL, left paracentral lobule; AG, right angular gyrus. [file Image_1.PDF]
