# Supplementary material for: Functional Connectivity and Networks Underlying Complex Tool-Use Movement in Assembly Workers: An fMRI Study
Source: Front Hum Neurosci. 2021 Oct 28;15:707502. doi: 10.3389/fnhum.2021.707502 (PMC8581229; doi:10.3389/fnhum.2021.707502)
Supplement: Supplementary Table 1 — Spearman’s correlation coefficient between identified functional connectivity and the scores of assembly task performance. [file Table_1.PDF]

Supplementary Material

Supplementary Table 1. Spearman's correlation coefficient between identified functional connectivity and the scores of assembly task performance

Variables	MTG and Crus II		SMA and IFGtri		PCL and AG	
	Rs	p-value	Rs	p-value	Rs	p-value
Time of assembly (sec)						
Total (n = 40)	**-0.33***	< 0.05	0.12	0.45	0.25	0.12
TW (n = 13)	0.06	0.83	0.35	0.24	-0.30	0.31
UTW (n = 27)	**0.41***	< 0.05	**0.66 ****	< 0.01	**-0.42 ***	< 0.05
Accuracy of assembly (%)						
Total (n = 40)	0.24	0.13	0.24	0.14	-0.24	0.13
TW (n = 13)	0.20	0.53	0.03	0.83	-0.52	0.13
UTW (n = 27)	0.11	0.59	0.24	0.23	0.03	0.87

Significant results are shown in bold font. * $p < 0.05$, ** $p < 0.01$

Rs = Spearman's rank correlation coefficient; Total = all participants; TW = trained workers; UTW = untrained workers; MTG = left middle temporal gyrus; Crus II = right cerebellum crus II; SMA = left supplementary motor area; IFGtri = pars triangularis of the right inferior frontal gyrus; PCL = left paracentral lobule; AG = right angular gyrus
